# Laparoscopic Excision of a Giant Parietal Peritoneal Lipoma: A Case Report

**DOI:** 10.7759/cureus.97751

**Published:** 2025-11-25

**Authors:** Jose A Suclla-Velasquez, Jimmy F Borda-Vera, Karla Rojas-Palacios

**Affiliations:** 1 Surgery, Servicio de Cirugía Hepatobiliar, Hospital Nacional Edgardo Rebagliati Martins, Lima, PER; 2 Surgery, Servicio Médico Quirúrgico, Hospital General Sicuani, Sicuani, PER; 3 General Practice, Servicio de Cirugía Hepatobiliar, Hospital Nacional Edgardo Rebagliati Martins, Lima, PER

**Keywords:** abdominal wall surgery, giant lipoma resection, laparoscopy, lipoma, parietal peritoneum

## Abstract

Lipomas are benign tumours derived from adipocytes, commonly found across the body, especially on the trunk and limbs. Although typically small, rare cases involve giant lipomas larger than 10 cm, often causing clinical symptoms. We report a case of a giant parietal peritoneal lipoma in a 45-year-old obese woman presenting with right upper abdominal pain. Imaging revealed a large, well-defined fatty mass in the abdominal wall, which was surgically excised laparoscopically. The specimen measured 18x14 cm and weighed 984 gm, with histology confirming a benign lipoma. The procedure was uncomplicated, with the patient discharged on day five and no recurrence over two years of follow-up. Only seven similar giant abdominal wall lipomas have been documented, with just one treated laparoscopically before. This case highlights the effectiveness and minimally invasive benefits of laparoscopic excision for large abdominal wall lipomas, which are rare but can present with symptoms mimicking other conditions.

## Introduction

Lipomas are benign tumours of adipocytes. Their origin is theorised to be displaced embryonic tissue or hyperproliferation [1,2]. Trauma is also associated with formation due to cytokines causing preadipocyte maturation. Genetics also play a role; some syndromes feature multiple lipomas, and the high mobility group AT-hook 2-lipoma preferred partner (HMGA2-LPP) fusion gene mutation is linked to these tumours [3]. As the most common mesenchymal tumours, they typically arise in the fourth to sixth decade of life [4]. They are usually painless and asymptomatic until growth affects cosmesis or causes symptoms based on location [2]. Lipomas must be primarily differentiated from atypical lipomatous tumours (ALT) and well-differentiated liposarcomas (WDLPS). Abdominal wall lipomas must also be distinguished from hibernomas and hemangiomas [5]. Therefore, image studies are mandatory for large masses. CT shows homogeneous fatty masses, and MRI shows them as isointense to subcutaneous fat. Thick septa or nodular/septal enhancement suggest malignancy [4,5]. Lipomas can be managed conservatively. Marginal excision is indicated for symptomatic cases or when appearance is affected [4]. While malignant transformation is not reported, recurrence occurs in 20% to 30% of cases due to incomplete excision [4]. We report a case of giant parietal peritoneal lipoma treated by laparoscopic excision. To our knowledge, only seven giant abdominal wall lipomas have been reported [1,6-11], and only one was excised laparoscopically [1].

## Case presentation

**Figure 1:**
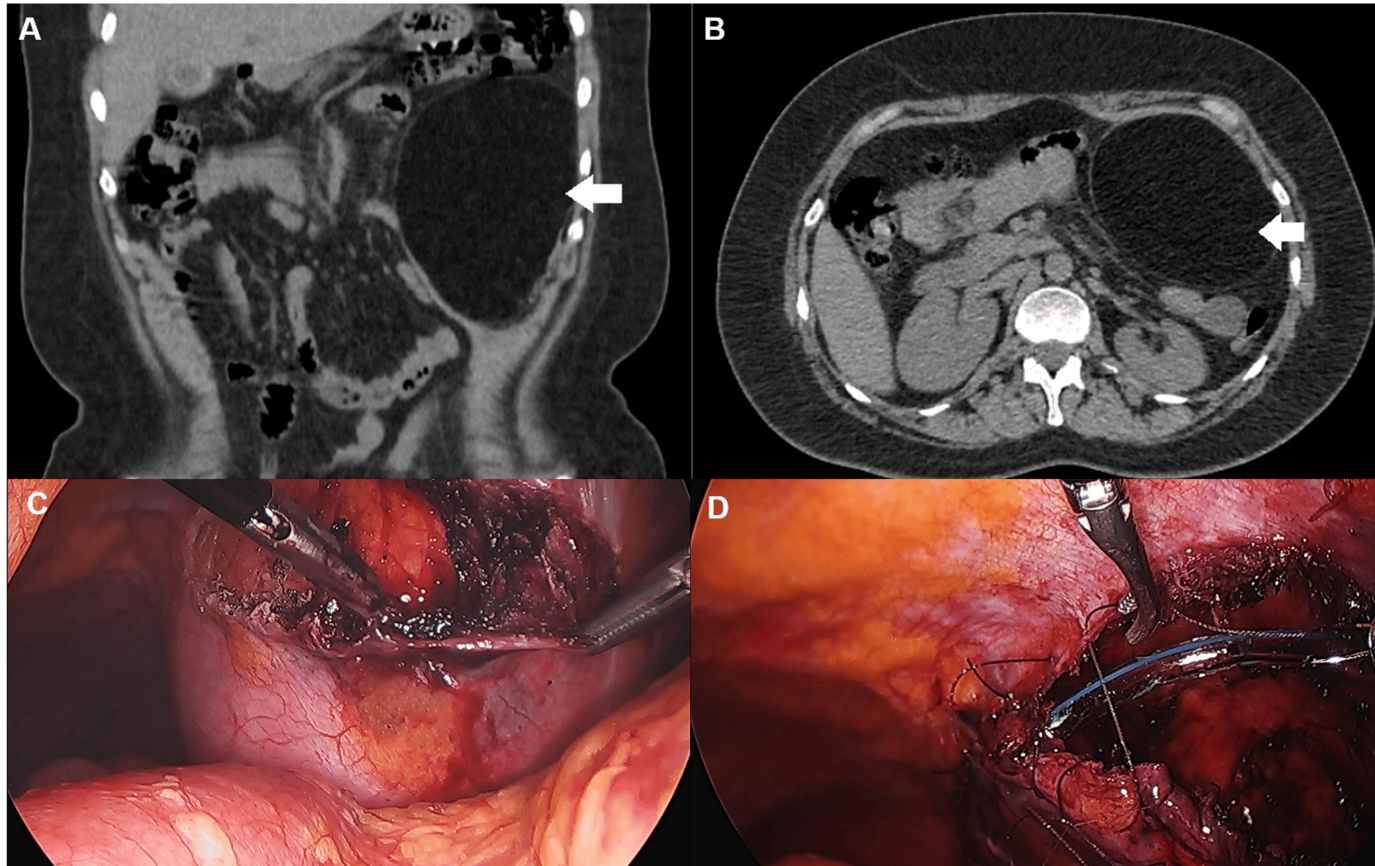
Giant parietal peritoneal lipoma (A, B) CT scan showing a large, well-defined, hypodense lesion in the parietal peritoneum (white arrows). The lesion appears homogeneous, with no significant contrast enhancement, consistent with a lipomatous tissue. (C, D) Laparoscopic images demonstrating the excision of the giant lipoma. The lesion is carefully dissected from the surrounding tissues, and the peritoneum is sutured after complete removal.

This is the case of an obese 45-year-old female without previous medical history who presented with a six-month history of right upper abdominal pain. Physical examination found a mass located in the left upper abdomen. An ultrasound showed chronic cholecystitis and a tumour located over the stomach and pancreas. A CT scan without and with contrast revealed a 13.6x10x9.3 cm, well-defined, fatty mass located in the abdominal wall, in front of the parietal peritoneum and the transverse abdominal muscle, in the left upper abdomen (Figures 1A, 1B). Contrast enhancement was not observed. Given these findings, ALT and WDLPS were ruled out. Other tumours, such as hibernomas and hemangiomas, were not considered due to their low frequency. Therefore, an abdominal wall lipoma was diagnosed, and surgery was offered because of its size and the patient's worry. However, the patient only accepted tumour excision and rejected cholecystectomy. We performed a laparoscopic tumour excision with parietal peritoneal closure. Four ports were inserted: two 10-mm ports, one in the umbilicus and the other in the left lower abdomen, and two 5-mm ports, one in the right upper abdomen and the other in the right lower abdomen. A mobile, soft mass in the parietal peritoneum was seen in the left upper abdomen (Figure 1C). Peritoneal incision was done, and the tumour was dissected from the peritoneum. Once the mass was excised completely, it was placed in a plastic bag and extracted through the extended umbilical port site. Finally, a closed suction drain was located, and peritoneal closure was done with absorbable suture (Figure 1D).

The resected specimen size was 18x14 cm, and the weight was 984 gm. The pathological diagnosis was reported to be a benign lipoma. The patient was discharged on postoperative day five. She has been followed up for two years now, and no recurrence has been noticed.

## Discussion

Lipomas are the most common mesenchymal tumours in the human body, composed of adipocytes. They have a slight male predominance and can be superficial or deep. Multiple lipomas are often associated with genetic syndromes [3]. Superficial lipomas primarily occur on the trunk, head, neck, and extremities. Abdominal lipomas are typically located in the omentum, mesentery, subserosa, and submucosa. Lipomas affecting the preperitoneal space of the abdominal wall are rare [7].

We identified 17 case reports of lipomas affecting the abdominal wall [1,2,6-20]. Of these, 12 presented with abdominal pain [8-10,12-20], one with urinary symptoms [1], four with an abdominal mass [6-8,11], and one was an incidental finding [2]. Our patient initially presented with right upper quadrant abdominal pain due to chronic cholecystitis, and the lipoma was incidentally discovered. Abdominal wall lipomas can also present with acute pain, potentially mimicking acute appendicitis when located in the right lower quadrant [12-14,16-18,20]. In these cases, where the tumours are usually no larger than 6 cm, torsion and ischemia are the cause of the pain.

Giant abdominal wall lipomas, larger than 10 cm or weighing over 1000 g, are less common. Only seven cases have been reported in the literature [1,6-11]. They have been described in association with abdominal pain, constipation, and urinary symptoms. The age of presentation ranges from 11 to 53 years, with a predominance in women. The largest reported lipoma weighed 3700 gm and measured 400 x 360 mm [10].

The diagnosis of lipomas is clinical when they are superficial. However, for abdominal wall lipomas, imaging studies such as ultrasound, magnetic resonance imaging, and computed tomography are often required for diagnosis [3]. Surgical treatment is the standard approach, with factors such as lesion size, anatomical location, symptoms, and patient comorbidities influencing determining the strategy [3].

## Conclusions

Although lipomas represent the most common mesenchymal tumours, giant abdominal wall lipomas are rare. Appropriate imaging studies should support diagnosis, and surgical excision remains the standard of care. Laparoscopic resection of such tumours has been previously described, and we report the second case documented in the literature. We consider that this minimally invasive approach, when technically feasible, offers significant advantages, including reduced abdominal wall trauma and faster postoperative recovery, facilitating an early return to daily activities.
